# Understanding the Interplay Between Premenstrual Dysphoric Disorder (PMDD) and Female Sexual Dysfunction (FSD)

**DOI:** 10.7759/cureus.62788

**Published:** 2024-06-20

**Authors:** Mahati Gollapudi, Angelica Thomas, Angelina Yogarajah, David Ospina, Jean C Daher, Aaliya Rahman, Lucia Santistevan, Ruby V Patel, Jeby Abraham, Sheethal G Oommen, Humza F Siddiqui

**Affiliations:** 1 Department of Medicine, Saba University School of Medicine, Caribbean, NLD; 2 Department of Internal Medicine, Anhui Medical University, Hefei, CHN; 3 Department of Family Medicine, Medical University of the Americas, Devens, USA; 4 Department of Internal Medicine, Universidad de los Andes, Bogotá, COL; 5 Department of Medicine, Lakeland Regional Health, Lakeland, USA; 6 Department of Medicine, Universidad de Ciencias Médicas Andrés Vesalio Guzman, San José, CRI; 7 Department of Internal Medicine, Dr. D. Y. Patil Medical College, Hospital and Research Centre, Pune, IND; 8 Department of Medicine, University of San Martín de Porres, Lima, PER; 9 Department of Surgery, Hemchandracharya North Gujarat University, Ahmedabad, IND; 10 Department of General Medicine, Yenepoya Medical College, Mangalore, IND; 11 Department of Psychiatry, University of Medicine and Pharmacy "Gr. T. Popa", Iași, ROU; 12 Department of Internal Medicine, Jinnah Sindh Medical University, Karachi, PAK

**Keywords:** combined oral contraceptive pills (cocps), selective serotonin receptor inhibitor (ssri), allopregnanolone, depression, sexual function, female sexual dysfunction (fsd), premenstrual dysphoric disorder (pmdd), premenstrual syndrome (pms)

## Abstract

Premenstrual dysphoric disorder (PMDD) is a severe variant of premenstrual syndrome (PMS), categorized as a mood disorder due to marked symptoms of depression and anxiety, compounded with severe physical symptoms. Female sexual dysfunction (FSD) can manifest as low libido, difficulty achieving sexual pleasure, and dyspareunia, causing functional and psychological distress. PMDD and FSD are globally prevalent conditions with postulated biological, psychological, and social associations between them. Nevertheless, sexual dysfunction in PMDD is an important aspect of women’s health that has been understudied and has notable methodological limitations. In this narrative review, we summarize the existing literature on sexual function in women with PMDD and PMS, specify the distinctions between PMDD and other general symptoms of PMS, highlight the significance of understanding sexual dysfunction in the female population, and outline some available therapeutic options. Studies show that women frequently experience debilitating sexual distress during the premenstrual phase; however, there is an essential need to formulate standardized tools for definite diagnosis. Selective serotonin re-uptake inhibitors (SSRIs) and combined oral contraceptive pills (COCPs) are approved medications for PMDD, while flibanserin and bremelanotide are effective in treating FSD. However, the potential effects of these treatment modalities on the two comorbid conditions render them inconclusive. Awareness of PMDD and FSD among clinicians and society can allow the implementation of targeted interventions to alleviate the suffering of women and enhance their quality of life.

## Introduction and background

Premenstrual dysphoric disorder (PMDD), a severe variant of premenstrual syndrome (PMS), is characterized by prominent mood symptoms including sadness, mood swings, anger, anxiety, irritability, decreased interest, fatigue, changes in appetite, and physical discomfort such as bloating and breast tenderness. These mood symptoms decrease in intensity or resolve completely after menstruation. An accurate diagnosis of PMDD requires prospective daily ratings for ≥2 cycles demonstrating a symptom-free period in the follicular phase. PMS and PMDD are triggered by hormonal events ensuing after ovulation, due to progesterone released by the ovary in the early, mid, or late luteal phase [1, 2]. The two neurotransmitter systems involved in the emergence of symptoms are the GABAergic and serotonergic systems. The metabolite of progesterone, allopregnanolone, formed by the corpus luteum of the ovary, readily passes the blood-brain barrier, and receptors for allopregnanolone are abundant in brain areas important for the regulation of emotions, cognition, and behavior. This metabolite diminishes GABA-mediated inhibition in the central nervous system by altering the configuration of the GABA receptor, making it immune to activation by binding at the neurosteroid binding site on the membrane of the receptor [3].

The potential influence of steroid hormones on psychological function leading to mood disorders has been explored in certain studies. Some methods of evaluating serotonergic activity in the brain have revealed insufficient serotonergic functioning, indicating a possible association between the appearance of PMS-like symptoms and decreased levels of serotonin [4]. In particular, abnormal regulation of the serotonin and allopregnanolone systems is implicated [5]. Serotonin reuptake inhibitors are considered first-line therapy, with second-line treatments including oral contraceptives containing ethinyl estradiol and drospirenone, other ovulation suppression methods, calcium, chasteberry, and cognitive-behavioral therapy [6, 7].

Approximately 3 to 8 percent of women of reproductive age (15 to 49 years) are thought to suffer from PMDD, which is considered a serious condition of PMS [8]. Premenstrual mood symptoms caused by PMDD can directly affect sexual function, and depression and anhedonia are among the main symptoms of PMDD [9, 10]. Not being able to enjoy or look forward to sexual activity can impact libido. Women with PMS are often unsatisfied with their body appearance and have concerns regarding self-efficacy, which can affect self-esteem and lead to poor sexual functioning [11]. Additionally, women with PMDD often struggle with mood regulation and lack of energy, further affecting sexual performance. More generally, stress from the mental and physical symptoms of PMDD can lead to poor sex and further aggravate the depression related to PMDD [10, 12].

**Figure 1:**
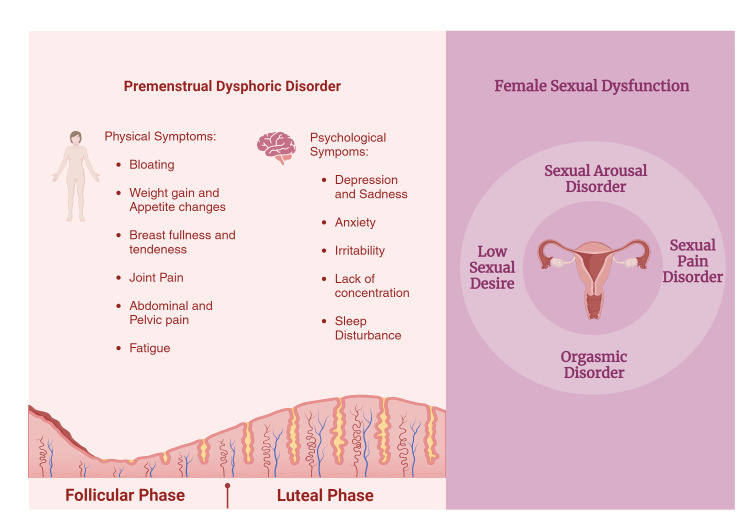
Premenstrual dysphoric disorder and female sexual dysfunction. Figure has been created using biorender.com

Understanding the association between PMDD and FSD is essential for numerous reasons. The burden of disease PMDD carries includes limitations on functional and sexual drive, disruption of partner relationships, female sexual dysfunction (FSD), and losses in work productivity. It affects the health-related quality of life, causing significant impairment in women’s overall functioning and perceived quality of life. The disability-adjusted life years (DALYs) lost due to this repeated cyclic disorder are comparable to those from commonly recognized conditions [13]. Despite their substantial impact, these issues are still under-recognized in large published epidemiological studies, and the association between PMDD and FSD is an important area in women's health that has been under-investigated and remains largely undiscovered [14, 15]. Elucidating the relationship between PMDD and FSD, and understanding the confounding factors that may link the two conditions can aid in implementing more effective ways to screen women for each illness and enhance formidable access to diagnosis and treatment. Figure 1 provides an overview of the article.

In this narrative review, we summarize the existing literature on sexual function in women with PMDD, revealing the findings and limitations in current literature. We analyze the reasons for the comorbidity of the two disorders and highlight the significance of female sexual health. We discuss the diagnosis, symptoms, and pathophysiology of PMDD and FSD, the psychological impacts, and therapeutic interventions for the two conditions, and the crucial impact they have on each other.

## Review

PMS and PMDD

Definition and Symptoms of PMS and PMDD

Premenstrual disorder consists of a range of symptoms that women of reproductive age experience during the luteal phase of the menstrual cycle. Depending on the type of symptoms, their severity, timing of occurrence, relationship to the menstrual cycle, and duration, these can be grouped into subsets of PMS, PMDD, or premenstrual exacerbation (PME) of another medical condition. These symptoms can be categorized as somatic, affective, and behavioral. Somatic symptoms include breast tenderness/swelling, joint pain, muscle pain, headaches, bloating, or weight gain. Affective symptoms include fatigue, lack of energy, anhedonia, difficulty concentrating, and changes in appetite and sleep. Mood symptoms include sadness, depression, feelings of worthlessness, irritability, anger, anxiety, or mood swings [16]. The American College of Obstetricians and Gynecologists (ACOG) defines PMS as having at least one affective or somatic symptom within the five days prior to menses, which must be relieved within four days of the onset of menses and must not recur until at least the 13th day of the cycle. The symptoms must have been experienced in the three previous cycles, should be reproducible in the subsequent two cycles prospectively, must not be due to any drugs or hormonal ingestion, and should negatively impact the patient's work, social, or academic performance [17, 18]. PMDD is considered a severe subset of PMS, with a stronger emphasis on mood symptoms. The Diagnostic and Statistical Manual of Mental Disorders, Fifth Edition (DSM-5) criteria define it as having at least five symptoms during the premenstrual period, of which at least one must be a mood symptom [18, 19].

Diagnosis of PMS and PMDD

Since the diagnosis of PMS/PMDD depends on the type of symptoms, their severity, timing of occurrence, relationship to the menstrual cycle, and duration, a thorough history and physical examination, including a gynecological examination, are imperative. Many tools aid in assessing the symptoms carefully, such as the Carolina Premenstrual Assessment Scoring System (C-PASS), The Daily Record of Severity of Problems (DRSP), The Menstrual Distress Questionnaire (MDQ), Premenstrual Symptom Screening Tool (PSST), Premenstrual Assessment Form (PAF), Daily Symptom Rating Scale (DSRS), and Calendar of Premenstrual Events (COPE) [19, 20]. However, there are drawbacks to using these tools for PMS/PMDD assessment. Each tool has its own unique set of elements used in symptom assessment. Some tools rely on retrospective data while others are prospective. Thus, the information obtained cannot be uniformly applied while attempting to diagnose PMS/PMDD. PMDD is a DSM-5 diagnosis, and any tool used to aid in its diagnosis is therefore expected to follow DSM-5 criteria. Unfortunately, some of these tools deviate from DSM-5 criteria, making it challenging to incorporate them into the decision-making process. In addition, these tools require recording symptoms on a daily basis for two months, demanding time and commitment, which makes these tools difficult for many patients to follow [18, 20]. Though a provisional diagnosis can be made on initial manifestation of symptoms, evaluating the symptoms for two consecutive menstrual cycles prospectively is needed to confirm the diagnosis of PMDD. C-PASS and DRSP are the two diagnostic tools that considerably aligns well with the DSM-5 criteria. This rigorous evaluation helps ensure that we are not misdiagnosing normal symptoms of menses as PMDD [19, 20].

Another essential point to consider is that PMS and PMDD are diagnoses of exclusion; as such, any organic (medical) or psychiatric conditions that can mimic symptoms of PMS/PMDD must be ruled out. Hematologic disorders such as anemia and leukemia, endocrinological disorders like thyroid disease, Cushing's syndrome, hyperprolactinemia, gastroenterological disorders, and gynecologic disorders such as irritable bowel syndrome and endometriosis, rheumatologic disorders, and pain syndromes such as chronic pain syndrome and fibromyalgia are some organic causes that mimic PMDD. Major depressive disorder, dysthymic disorder, panic disorder, generalized anxiety disorder, personality disorder, and seasonal affective disorder are some psychiatric conditions that exacerbate luteal phase symptoms [16, 19]. Thus, careful investigation is needed to rule out other mimickers of PMDD, and further tests such as complete blood count (CBC), follicle-stimulating hormone (FSH), estradiol, thyroid-stimulating hormone (TSH), prolactin, cortisol, and breast and pelvic imaging may be needed depending on the scenario [21, 22, 23, 24]. Moreover, since cigarette smoking, alcohol consumption, substance use, intimate partner violence, past trauma, and obesity are risk factors for PMDD, a thorough social history is also warranted [16, 21, 24].

A history of childhood maltreatment has been found to be linked with depression among adolescents. Specifically, individuals who have experienced significant emotional trauma are more likely to exhibit heightened depressive symptoms in response to stressful life events. Furthermore, instances of emotional and sexual abuse within parent-child relationships have been associated with disturbances in the development of emotional regulation mechanisms, thus contributing to the manifestation of mental health disorders [25]. In women with PMDD without existing PTSD or other psychological issues and not using medication, a previous history of abuse is associated with changes in sympathetic function. These changes are apparent in differences in resting levels of norepinephrine, heart rate, and responsiveness to mental stress [26].

Prevalence of PMS and PMDD

Although different articles mention varying prevalence rates, many studies state that as high as 70-90% of women of reproductive age (15 to 49 years) experience some level of premenstrual symptoms [16, 19, 20, 21, 22]. Of these, about 30-40% of women have reported PMS symptoms, while 3-9% have met the criteria for a diagnosis of PMDD [16, 17, 20].

The peak age for presenting in healthcare settings for PMDD is in the late 20s or 30s, although symptoms may initiate as early as the teenage years [27, 28, 29]. One study revealed that the average age at which a female patient was clinically diagnosed with PMDD was 35 years (range: 20 to 45 years), although the average age of onset of symptoms was 15 years (range: 11 to 38 years) [30]. A study performed on adolescent schoolgirls revealed a prevalence rate of 41.7%, 53%, and 5.1% among age groups 14 to 15 years, 16 to 17 years, and 18 to 19 years, respectively [31]. Similarly, PMDD was found in 4.8% of the subjects aged between 11 to 20 years in another study [32]. A prevalence of 37% was reported among university students aged 19 to 28 years, slightly higher than the studies that delineated a prevalence of 6 to 18% in a similar patient cohort [33]. The diagnosis of PMDD was established among 6.4% of female patients aged between 36 to 44 years. 2.7% of patients were diagnosed with PMDD with no previous history of depression [34].

Levels of estrogen were found to be substantially higher among female teenagers (15 to 19 years) who reported premenstrual symptoms compared to those free of symptoms (148.3 pg/ml vs 98.0 pg/ml, p < 0.05) [35]. FSH and luteinizing hormone (LH) levels were significantly lower, while estrogen and progesterone levels were slightly higher in the late luteal (LL) phase among patients with PMS aged 21 to 43 years compared to healthy individuals without symptoms [36]. A study conducted on young adults (mean age of 25) showed that significantly lower levels of estrogen and higher levels of progesterone in the early luteal (EL) phase positively correlated with the occurrence and severity of symptoms of PMDD [37]. The age of menarche was found to be a potential risk factor for the development of PMDD among young adults (25.7 ± 3.5 years). Patients with an early age of menarche (mean 11.6 years) had a higher prevalence of PMDD, while those with late menarche (mean 13.9 years) had comparatively lower frequencies of PMDD [38]. Data regarding the progression of symptoms of PMDD is conflicting. Some studies highlight higher severity of symptoms in older age groups among comparable ages. Conversely, other studies have shown that older age groups are related to less severe symptoms. Several factors including levels of stress, exercise, sleep, and diet may plausibly contribute to the severity of PMDD symptoms regardless of age [39, 40].

Pathophysiology of PMS and PMDD

Extensive research has been conducted to find the possible causes of PMS and PMDD. However, no single theory has been universally accepted as the pathophysiological cause of PMS or PMDD, and the conditions are believed to be multifactorial. The correlation of mood changes with hormonal changes in the menstrual cycle strongly supports the role of the hypothalamic-pituitary-gonadal axis and ovarian sex steroids in PMDD. However, studies have not found any differences in the level of sex hormones between PMDD patients and asymptomatic females. Instead, studies have shown that PMDD patients have altered sensitivity to the normal fluctuation of these hormones during the menstrual cycle [16, 19, 21, 22, 23, 24]. Researchers have proven through various methods that the interaction of sex steroids such as progesterone, allopregnanolone, estrogen, and testosterone, with central nervous system-mediated neurotransmitters such as serotonin, GABA, dopamine, norepinephrine, and acetylcholine, is possibly the cause of the physical and psychological symptoms experienced in PMDD [16, 19, 21, 22, 23, 24]. Some studies have found a possible role for inflammation and immune activation as changes in c-reactive protein (CRP), interleukin-6 (IL-6), and tumor necrosis factor-alpha (TNF-alpha) have been observed during the symptomatic period [24]. Relationships with prolactin levels, insulin resistance, glucose metabolism alterations, electrolyte abnormalities, genetics, stress, and trauma, including both physical abuse and sexual abuse, have been seen in patients with PMDD [21, 24]. Among these multiple theories, serotonergic dysregulation seems to be the most plausible explanation at present. The fact that treatment of PMDD with selective serotonin re-uptake inhibitors (SSRIs) results in symptomatic relief of both physical and affective symptoms further strengthens the role of serotonin in PMDD [16, 19, 21, 22, 23, 24].

Female sexual dysfunction (FSD)

Understanding FSD

FSD is a common but often under-recognized condition that can significantly impact the quality of life of affected individuals [41, 42]. Rates of incidence and prevalence differ but have been documented to be between 30 and 50% in women. These figures are likely lower than the actual numbers due to the enduring societal taboo linked to admitting female sexual distress. This stigma may have contributed to a scarcity of literature on FSD [43]. FSD encompasses a range of sexual problems or difficulties that lead to personal distress. According to the fifth edition of the Diagnostic and Statistical Manual of Mental Disorders, 'Sexual dysfunctions are a heterogeneous group of disorders that are typically characterized by a clinically significant disturbance in a person’s ability to respond sexually or to experience sexual pleasure; an individual may have several sexual dysfunctions at the same time' [42, 44].

The symptoms of FSD can be diverse, encompassing aspects such as sexual desire, arousal, orgasmic response, and potential discomfort [43, 45]. In terms of desire, women with FSD may experience a lack of interest in sexual activity, reduced libido, or aversion to sexual contact. Arousal challenges may present as difficulty in becoming sexually aroused or in sustaining arousal during sexual activity [46]. Orgasmic difficulties can include the inability to achieve orgasm, delayed orgasm, or experiencing an unsatisfactory orgasm. Painful sexual intercourse, referred to as dyspareunia, is a prevalent symptom of FSD with various underlying physical and psychological factors [47, 48, 49].

Classification Systems for FSD

The Diagnostic and Statistical Manual of Mental Disorders, 5th edition, Text Revision (DSM-5-TR), provides a comprehensive classification system for FSD. It categorizes FSD into different subtypes, such as female sexual interest/arousal disorder, female orgasmic disorder, and genito-pelvic pain/penetration disorder. Symptoms must persist for a minimum of six months and result in considerable distress [14, 50]. One of the advantages of the DSM-5-TR classification is its detailed descriptions of each subtype, which can aid in accurate diagnosis and treatment for a large population [47]. However, a major limitation is the high rate of false negatives, as the system may fail to identify all patients with sexual dysfunction. Combining two separate diagnostic categories, for instance, hypoactive sexual desire disorder (HSDD) and female sexual arousal disorder (FSAD), into a single category reduces the specificity and precision of the classification, potentially leading to misdiagnosis and inadequate treatment for some individuals without definite symptoms [51].

In the International Classification of Disease-11 (ICD-11), FSD is classified as hypoactive sexual desire dysfunction, sexual arousal dysfunction, or orgasmic dysfunction, comparable to the DSM-5-TR’s classifications [11, 52]. Women experiencing unexplained pain are categorized under the 'Conditions Related to Sexual Health' chapter with sexual pain-penetration disorder. Dyspareunia, described as persistent genital pain before, during, or after intercourse or penetration, along with vulvodynia, and pelvic pain, are included in the Diseases of the Genitourinary System chapter and may lead to sexual dysfunctions [52].

To be classified as FSD in ICD-11, symptoms must cause debilitating distress, continue to be present for a few months, and occur regularly. The ICD-11 also differentiates between chronic and acquired FSD and whether the difficulties occur in all sexual aspects or only in specific circumstances [52, 53].

The International Council for Sexual Medicine (ICSM) and the International Society for the Study of Women's Sexual Health (ISSWSH) also developed their own classification systems for FSD. These systems distinguish themselves from the DSM-5-TR by seeking to provide a more thorough understanding of FSD. They achieve this by differentiating between female sexual desire and arousal disorders and providing detailed explanations of subtypes of arousal issues. The categorization of FSD into primary and secondary disorders is also employed, where primary disorders entail lifelong sexual difficulties, while secondary disorders manifest after a period of normal sexual function. This classification system adopts a comprehensive approach that takes into account both physical and psychological factors contributing to sexual dysfunction in women [14, 53, 54].

Subtypes of arousal problems encompass sexual interest-desire dysfunctions, genital arousal disorder, subjective arousal disorder, combined genital and subjective arousal disorder, persistent genital arousal disorder, dyspareunia, vaginismus, a unisex definition of sexual aversion, and women’s orgasmic disorder [53, 54]. According to the ISSWSH and ICSM terminology, FSD includes HSDD, FSAD, persistent genital arousal disorder, female orgasmic disorder, and genito-pelvic pain/penetration disorder [14]. Additionally, ICSM and ISSWSH focus on the potential etiologies and risk factors for FSDs, offering a more holistic perspective on female sexual health [47]. Each classification system for FSD has its own strengths and limitations, and the preference regarding its application depends on the specific needs of clinicians and researchers in different perspectives.

Diagnosis of FSD

Diagnosing FSD may be complex due to its diverse causes, including anatomical, psychological, hormonal, or medication-induced factors. The clinician must first initiate a conversation with the patient and obtain a detailed obstetric and gynecologic medical history, including histories of sexual abuse, sexually transmitted diseases, bowel or urinary complaints, and surgeries [55]. Women who report dyspareunia may experience pain on deep penetration, raising suspicion for pathologies such as endometriosis and leiomyomas. Conversely, some patients may complain of superficial pain and itchiness. If vulvar or vaginal skin abnormalities are noted during the physical examination, conditions like lichen sclerosus or vaginal atrophy, attributable to hormonal deficiencies, should be suspected. The observation of vaginal discharge during examination suggests a possible bacterial or fungal infection. Another cause of sexual dysfunction that may be observed during a physical exam is pelvic organ prolapse, typically diagnosed visually [56]. When a patient presents with superficial pain combined with pelvic floor muscle stiffness, it indicates vaginismus. Decreased sexual desire reported by women may be attributable to hormonal deficiencies or external factors. When a patient presents with decreased sexual desire, further evaluation of symptoms is necessary to elucidate a diagnosis. An enlarged thyroid on examination may raise suspicion of hypothyroidism. If this is accompanied by galactorrhea, further workup to rule out a prolactinoma must be conducted. Diabetes or a neurological condition should be suspected if FSD presents with concomitant neuropathy. Decreased libido associated with pallor usually indicates anemia. Lastly, if the patient presents with decreased sexual desire in conjunction with irregular peripheral pulses or blood pressure, it is essential to rule out a vascular etiology [57].

Although physical examinations are normal in most cases, a complete pelvic examination can help establish a diagnosis and reassure the patient that she has a treatable pathology that can lead to the resolution of symptoms. The pelvic examination begins with a visual examination of the vulva, labia majora, and labia minora. Externally, it starts with the outer genitalia, usually using a gentle vaginal swab [56]. This superficial examination may reveal pathologies such as condylomas, vulvodynia, vulvovaginitis, infectious lesions, or atrophy [58]. Following this superficial examination, the internal examination of the pelvis commences with a manual examination of the pelvic floor muscles, bladder, uterus, anus, and adnexa. This may reveal unusual tenderness or pelvic masses causing discomfort. Conventionally, pelvic floor muscles should not be tender to palpation, and they should relax and contract voluntarily during an internal pelvic examination [59].

Causes of FSD

The etiology of FSD is multifactorial, with many patients reporting concerns across a variety of symptom complexes [44, 45]. FSD can be influenced by psychological factors such as stress, depression, anxiety, past sexual trauma, low self-esteem, and concerns about body image. Medical elements such as chronic illnesses, hormonal imbalances, menopause, specific medications, and pelvic area surgeries may also contribute to the condition [41, 42, 60, 61].

Relationship challenges, including partner-related issues and lack of intimacy or communication difficulties, could also be factors. Societal expectations, cultural norms, and financial pressures are some of the social, political, and economic elements that might impact FSD [61, 62]. Physical considerations encompass pain during intercourse, inadequate vaginal lubrication, and other conditions affecting sexual arousal or pleasure [42, 44]. Additionally, challenges in relationships, cultural values, and societal standards related to sexuality can influence a woman's sexual well-being [48].

It is crucial to highlight that these factors frequently intersect, and treating FSD typically involves a comprehensive approach that encompasses both the physical and mental components of sexual function [41, 49].

Pathophysiology of FSD

As discussed above, female sexual function is intricate, encompassing psychological, relational, and physiological elements. Neurohormonal influence, the central nervous system, and the peripheral nervous system also contribute largely to the dysfunction [46, 62].

Hormones such as estrogen, which influences sexual arousal and desire, and testosterone, which impacts sexual desire, along with dehydroepiandrosterone, with an unclear understanding of its effect on sexual function-norepinephrine, serotonin (affecting sexual arousal), dopamine (influencing sexual desire and arousal), and nitric oxide, which may affect clitoral vasocongestion, play key roles [45, 46].

Vascular elements that affect blood flow to the reproductive organs may play a role in experiencing arousal challenges, as well as other indications of FSD [45, 62]. Among peri- and postmenopausal women, the steady decline in circulating levels of estrogen and testosterone and other related hormones that occur with age, which may begin as early as age 20, are major contributing factors affecting female sexual function [44, 60]. For example, diminishing estrogen levels lead to vaginal epithelial thinning, decreased vasocongestion, and reduced lubrication during sexual arousal, resulting in pain and distress during intercourse [44, 63, 64].

Since sexual well-being is regarded as a significant component of both physical and mental health, experiencing PMS can lead to various physical and psychological symptoms that may have negative implications for women's sexual functioning. Moreover, individuals with PMS may also experience heightened personal distress related to sexuality [62, 65]. Overall, the pathophysiology of FSD is complex and involves a combination of vascular, hormonal, psychological, and neurological factors [60-65].

Association between PMS/PMDD and FSD

PMS is considered an umbrella term for various mood and physical symptoms that occur during the luteal phase and typically resolve a few days after menstruation. PMDD is associated with greater psychosocial impairment and more prominent mood symptoms than PMS. Different sexual issues in women, including low libido, difficulty reaching or achieving orgasm, and dyspareunia, can be categorized as FSD.

Some studies have highlighted the association of PMS and PMDD with FSD. The most recent study evaluating a link between PMS and FSD, a 2021 cross-sectional study by Conzatti et al., found lower scores on the Female Sexual Function Index during the luteal phase compared to the follicular phase but did not find a consistent association between PMS/PMDD and FSD using validated scores for both PMS (Premenstrual Symptoms Screening Tool - PSST) and FSD (Female Sexual Function Index - FSFI) over at least one menstrual cycle. The study included women aged 18 to 45. All participants were required to fill out an initial form to rule out common mental disorders (Primary Care Evaluation of Mental Disorders - PRIME MD). Other exclusion criteria included pregnancy, use of medications that may impact sexual function, such as antipsychotics, benzodiazepines, and antidepressants, sexual inactivity, and continuous use of hormonal contraception. The FSFI scores ranged from 2 to 36 and evaluated sexual function across six categories including desire, arousal, lubrication, orgasm, satisfaction, and pain, with each item scored from 0 to 5. A score of 26.55 or less was considered diagnostic of FSD [66].

Wood et al. evaluated various psychosocial factors against menstrual characteristics in an early study performed in 1977. No subclassification between PMS or PMDD was established. The study reported that women who experienced difficulties with their sex life also more frequently experienced menstrual pain and premenstrual stress, although no specific values were provided [67]. In 1991, Winter et al. conducted a study in 26 women previously diagnosed with PMS and 26 women without PMS, administering three questionnaires that evaluated sexual function and satisfaction: the Index of Marital Satisfactions (IMS), Index of Sexual Satisfaction (ISS), and Index of Family Relations (IFR). Scores on these surveys ranged from 0 to 100, with higher scores indicating a clinically significant problem in marital, sexual, or family relationships; a cutoff value of 30 or more was established for each survey. The presence or absence of PMS was subjectively determined by a previous diagnosis established by a nurse practitioner or gynecologist. Results showed that women with PMS scored higher on all three surveys compared to non-PMS controls, although few cases reached scores above the cutoff value for clinical significance [68].

The largest study, published by Nowosielski et al. in 2010, involved a sample of 1540 Polish women. PMS was diagnosed by the presence of at least one symptom such as breast swelling or tenderness, fatigue, bloating, lack of energy, change in appetite, sleeping distress, headache, impulsivity, mood lability, depressed mood, anxiety, agitation, social withdrawal, feeling of loss of control, decreased concentration, and irritability, during the premenstrual phase that resolved following menstruation. The diagnosis of PMDD was made using author-developed questionnaires that evaluated the DSM-IV criteria for PMDD, confirmed by symptom recording for two menstrual cycles. The study did not include a validated tool for evaluating FSD and only measured specific risk factors for FSD. No significant differences in risk factors for FSD were found in women with or without the diagnosis of both PMS or PMDD, although 26.65% of women overall reported sexual distress [69].

A Turkish study by Ilhan et al. demonstrated a higher degree of both sexual difficulty and sexual distress in women with PMS by prospectively evaluating PMS symptoms using the Daily Record of Severity of Problems (DRSP) score, and FSD symptoms using the Female Sexual Function Index (FSFI) with a cutoff value of 26.55 or less for the diagnosis of FSD in a sample of 286 women, including 143 women with PMS as well as 143 without PMS. PMS was confirmed by measuring the DRSP score over two menstrual cycles. Results showed that 77.6% of women categorized as PMS positive had sexual concerns as opposed to only 27.3% of women without PMS. Sexual dysfunction and distress were present in 51.7% of women with PMS and in 24.5% of women without PMS [65]. Yang et al. conducted a secondary analysis of an online survey study for the Premenstrual Symptoms Impact Survey (PMSIS). Higher scores on the PMSIS were directly proportional to greater functional limitations affecting health-related quality of life (HRQOL). A significantly higher percentage of women with PMS and PMDD reported a negative impact on sexual drive, 67.5% and 73.3% respectively [70]. Table 1 summarizes all studies associating PMS/PMDD and FSD.

**Table 1:** Association between FSD and PMS/PMDD. DRSP: Daily Record of Severity of Problems; PSST: Premenstrual Symptoms Screen Tool; FSFI: Female Sexual Function Index; NA: Not Applicable; NP: Nurse Practitioner; IMS: Index of Marital Satisfactions; ISS: Index of Sexual Satisfaction; IFR: Index of Family Relations; PMSIS: Premenstrual Symptoms Impact Survey; OB/GYN: Obstetrics and Gynecology; PMS: Premenstrual Syndrome; PMDD: Premenstrual Dysphoric Disorder; DSM-IV: Diagnostic and Statistical Manual of Mental Disorders-IV, FSD: Female Sexual Dysfunction.

Author Name (Year)	Article Tile	Type of Study Design	No. of Menstrual Cycles	Questionnaire for PMDD	Questionnaire for FSD	No. of Participants (n)	Conclusion
Conzatti et al (2021) [66].	Premenstrual syndrome and female sexual function	Cross-sectional study	≥ 1	PSST & DRSP	FSFI	69 women in PMS group, 52 women in control group	No difference in the FSF between groups in both cycle phases
Ilhan et al (2017) [65].	Premenstrual Syndrome Is Associated with a Higher Frequency of Female Sexual Difficulty and Sexual Distress	Prospective	2	DRSP	FSFI	286	Sexual concerns and sexuality-related personal distress rates were higher in PMS positive group (P <0.05).
Nowosielski et al (2010) [69].	Sexual satisfaction in females with premenstrual symptoms	Prospective Population Study	2	Author-designed questionnaire using DSM-IV criteria for PMDD	Author-designed questionnaire evaluating sexual behaviors	1540	Women from the PMS group were less sexually satisfied than controls, and reported more sexual distress. (P=0.001)
Yang et al (2010) [70].	Interpreting Premenstrual Symptoms Impact Survey scores using outcomes in health-related quality of life and sexual drive impact	Retrospective	NA	PMSIS	A single item evaluating “marked change in sex drive”	112 women deemed “at risk” for PMS, 780 women “not at risk” for PMS or PMDD	More women with PMS and PMDD reported sexual drive impact than women without PMS
Winter et al (1991) [68].	Dispelling myths: a study of PMS and relationship satisfaction	Cross-Sectional Study	NA	Diagnosis of PMS / PMDD previously given by NP or OB/GYN	IMS, ISS, IFR	26 women diagnosed with PMS, 26 non-PMS controls	Patients with PMS scored higher on all three scores when compared to non-PMS controls
Wood et al (1979) [67].	Social and Psychological Factors in Relation to Premenstrual Tension and Menstrual Pain	Retrospective	NA	Self-reporting for “premenstrual tension” or “menstrual pain”	NA	699	Menstrual pain and premenstrual tension were more frequent in women that reported issues with their sex life

PMDD can seriously impact women’s wellness, including their sexual health. Recent data also suggest a high prevalence of sexual dysfunction in women with PMDD, broadly associated with both psychological and physiological symptoms, though causation has not been proven. Similarly, although causality is yet to be defined, the emotional storms that PMDD brings, such as mood changes and irritability, may lead to deterioration in relationship dynamics, providing a clue as to why sexual dysfunction is co-occurring with PMDD [14, 71].

Since PMDD follows a cyclical pattern, shifts in estrogen and progesterone can influence sexual function and response. Estrogen levels drop during the premenstrual period, which can manifest as low libido and lack of lubrication. Estrogen decline can also contribute to reduced intensity of orgasm or difficulty in achieving climax. During the luteal phase, progesterone increases fatigue and lethargy. As progesterone levels rise, those with PMDD might experience a decline in sexual desire and motivation, which can result in decreased sexual satisfaction [4].

Hormones and neurotransmitters in both the brain and peripheral body can combine to interfere with sexual function. Serotonin, the neurotransmitter that regulates mood, is dysregulated in PMDD, and emotional symptoms could potentially impact sexual function. Low serotonin correlates with low desire and difficulty climaxing. To add insult to injury, selective serotonin reuptake inhibitors (SSRIs), which are commonly prescribed for PMDD, can lead to lower libido, difficulties climaxing, and genital numbing [9, 72]. These challenges make addressing PMDD a complex problem.

Physical symptoms can have direct effects on sexual function as well. Bloating, a symptom associated with PMDD, can result in discomfort, making sexual activity painful and unpleasurable for women. Breast tenderness, another common symptom associated with PMDD, can make intercourse uncomfortable and painful if direct stimulation or pressure is involved. Simply touching sore breast tissue can be painful, and the discomfort associated with this can make seeking out or even initiating sex difficult. PMDD may also manifest with intense fatigue. Sexual function can be impacted by this, as participating in sex can be difficult when one feels very tired. Fatigue may also lead to difficulties in arousal and maintaining stamina during sex. It is important to acknowledge and address these physical symptoms to support individuals with PMDD in maintaining a satisfying and fulfilling sex life [14].

Approved therapeutics for FSD and PMDD

The landscape of treatment for FSD and PMDD is evolving, with new etiologies and contributing factors being identified. Despite several therapies under investigation showing promising results, the number of agents approved by the FDA for these indications remains limited. On the FSD spectrum, only HSDD and vulvovaginal atrophy (VVA) with resulting dyspareunia have approved therapeutic options.

For HSDD, the approved therapies are flibanserin and bremelanotide [73, 74, 75]. Flibanserin is a serotonin 5-HT1A agonist and 5-HT2A antagonist that reduces the inhibitory effect of serotonin and increases the excitatory effect of dopamine. By disinhibiting noradrenergic neurons in the locus coeruleus, it increases norepinephrine concentrations in the prefrontal cortex. Flibanserin is orally administered as a 100 mg tablet at bedtime to minimize the potential side effect of drowsiness. Other important side effects include syncope and hypotension. It is not safe for persons with any kind of hepatic impairment or for the geriatric population. Medication review and patient counseling are necessary before prescribing flibanserin, as it can interact with CYP3A4 inhibitors and alcohol [73, 75]. Bremelanotide is a melanocortin receptor agonist that modulates the neurotransmitter pathways of sexual desire and arousal in the prefrontal cortex and limbic system [74]. It can be administered intranasally or via subcutaneous injection. 1.75 mg of bremelanotide is injected subcutaneously in the thigh or abdomen at least 45 minutes before sexual activity. This route offers complete bioavailability and has fewer adverse effects. Dosing is once every 24 hours, and no more than 8 doses should be taken in 1 month. Adverse effects include transient hypertension, nausea, headache, and hyperpigmentation. Contraindications include uncontrolled hypertension, cardiovascular disease, and concurrent use of indomethacin or naltrexone [75]. Topical estradiol, ospemifene, and synthetic dehydroepiandrosterone (DHEA) have been approved to treat VVA and dyspareunia; however, this diagnosis only applies to the postmenopausal population and is therefore not in the scope of this review [76, 77, 78].

For PMDD, the current FDA-approved treatments are fluoxetine, sertraline, paroxetine, and drospirenone/ethinyl estradiol [79]. Women with PMDD are known to have fewer serotonin transporter receptors, and serotonergic antidepressants, particularly selective serotonin reuptake inhibitors (SSRIs), can improve symptoms. Fluoxetine, sertraline, and paroxetine fall under this class, and SSRI therapy is now considered the first-line treatment for PMDD. Interestingly, SSRIs start showing effectiveness within a few days of initiating treatment. Therefore, they can be taken intermittently from ovulation until day 1 of menstruation, during the luteal phase from symptom onset until day 1 of menstruation, or continuously. SSRIs may work by indirectly modulating the function of GABAA receptors [80]. SSRI therapy has been shown to improve the psychological symptoms of PMDD more than the physical symptoms but does not appear to reduce headaches associated with PMDD [19, 79]. Adverse effects of SSRIs include sexual dysfunction and weight gain, which may contribute to FSD. Concurrent treatment with bupropion may mitigate treatment-emergent FSD [81]. Drospirenone 3 mg/ethinyl estradiol 20 μg is a combined oral contraceptive pill (COCP) formulation approved for PMDD. Since the fluctuations in gonadal steroids during the menstrual cycle are responsible for the emergence of symptoms, replacing them with a steady state of exogenous steroids provides relief. Active pills are given once daily for 24 days, followed by placebo for 4 days. It has been shown to improve both the emotional and physical symptoms of PMDD [82]. Intermenstrual bleeding, breast pain, and nausea are common side effects, and the risk of venous thromboembolism is higher with drospirenone than with other progestins [19, 79, 80].

Understanding the Effects of Oral Contraceptive Pills (OCPs) on FSD and PMDD

The ovarian steroid hormones estradiol, testosterone, and progesterone play crucial roles in female physiology, and OCPs modulate them to prevent pregnancy [83]. OCPs work by suppressing ovulation and preventing the fluctuations in ovarian steroids that occur during the menstrual cycle [79]. Combined OCPs (COCPs) include an estrogen component and a progestogen component, while progestin-only OCPs (POPs) contain only a progestogen component [84]. Earlier formulations of COCPs contained high doses of estrogens, but due to their adverse effects, newer formulations with lower estrogen doses have been developed. These formulations have evolved, with mestranol being replaced by ethinyl estradiol (EE) or 17β-estradiol (E2), and older testosterone-derived progestins, which had more androgenic side effects, being replaced by progesterone- and spironolactone-derived progestins (e.g., drospirenone, chlormadinone acetate). Most modern COCP formulations contain 35 μg or less of EE. The POP used in the United States contains 35 μg of norethindrone [85, 86].

COCPs can be administered in a standard dosage cycle for 21 days, followed by a placebo for 7 days, to mimic the 28-day menstrual cycle and cause withdrawal bleeding during the placebo week. Modified regimens, such as extended-cycle formulations, only have one placebo week every 12 weeks [85]. Monophasic OCPs maintain the same hormone dose per day, while multiphasic OCPs vary hormone levels over the cycle. Common adverse effects of COCPs include weight gain, breast tenderness, and nausea, while major adverse effects include venous thromboembolism, stroke, and myocardial infarction. POPs can be prescribed to women at risk of these major adverse events [85, 86].

Research into FSD has historically been limited by the number and complexity of variables affecting female sexuality, and attempts to identify predictors of FSD have met with challenges such as sociocultural differences and patients' reticence to confide in medical professionals [87]. Sexual functioning in otherwise healthy women varies based on age, individual biology, reproductive status, menstrual phase, and exogenous hormones. The DSM-V classifies FSD into dysfunctions resulting from pain/penetration, lack of interest/arousal, and inability to achieve orgasm [88].

OCP use has been linked to changes in female sexual function. On one hand, they can potentially improve function by reducing the fear of pregnancy and physical symptoms of gynecologic problems like dysmenorrhea and hirsutism. On the other hand, since they reduce both androgen and estrogen concentrations, they have been associated with FSD. Theoretically, low estrogen concentrations can lead to vulvovaginal atrophy, although there is insufficient data to link OCPs to this condition directly. In most cases, COCPs are associated with no change in sexual function, though a minority of women report improvement or worsening of sexual function related to desire, arousal, orgasm, and response [86]. Among women who develop sexual distress, HSDD is most commonly reported [89]. Studies that have looked into the specific sexual side effects of the drospirenone 3 mg/ethinyl estradiol 20 μg COCP have found conflicting findings, with reports of both improvement and worsening in female sexual function [90, 91]. There is a notable lack of research into therapeutic options for women who have both FSD and PMDD, especially for those who cannot or are not willing to take COCPs. Given the inconclusive nature of the studies that have looked into how COCP use affects female sexual functioning, in some women with PMDD who take COCPs, treatment-emergent FSD could very well contribute to worsening of mood and overall well-being. This represents an unmet need that warrants further research and exploration of alternative therapies. Another potential area for expansion in FSD literature is that most existing research and evaluation tools are only applicable to heterosexual encounters [84-91].

One of the potential etiologies of PMDD is altered sensitivity of the central nervous system to neuroactive steroid hormones. Allopregnanolone (ALLO), a metabolite of progesterone, is an allosteric modulator of the gamma-aminobutyric acid (GABA)-A receptor (GABAA-R). It is hypothesized that the symptoms of PMDD, and their timing of onset, may be a reflection of insufficient GABAA-R sensitivity to ALLO [90]. COCPs containing 3 mg of drospirenone and 20 μg of EE have been found to reduce symptoms of PMDD and are FDA approved for this indication. A 4-day placebo period is more effective than the standard 7-day interval [91]. For women with PMDD who take other COCPs, monophasic pills are generally recommended to avoid hormone fluctuations that can contribute to worsening mood [92]. Progesterone on its own has not been found effective to treat PMDD [93]. COCPs can cause mood deterioration in women with pre-existing mood, anxiety, or eating disorders, and they are not suitable for women with PMDD who plan to conceive [91]. Further research is needed in this domain to explore the differences in how women respond to changes in ALLO and to identify other biological factors contributing to the development of PMDD.

Generally, SSRIs are considered the gold standard for effectively controlling PMDD symptoms. However, many women decline this treatment option due to the possible negative stigma associated with psychoactive drugs. Most women who suffer from PMDD are of childbearing age, and OCPs maintain hormone levels as well as provide contraceptive protection. Early studies completed in the field disputed the efficacy of OCP use as a treatment for PMDD. A nested-cohort study was completed with 976 premenopausal women in Massachusetts, where 12.3% of them reported improvement of symptoms, however, 16.3% reported OCP-related mood decline. This study reported that OCPs do not influence mood in women despite improved somatic symptoms. Similarly, another cross-sectional study in Australia had 181 women answer questionnaires regarding their mood, and no association was found linking OCP use to the incidence or severity of PMDD. OCPs did show improvement in somatic symptoms presented by PMDD, however, the mood component was not alleviated, leading to the dispute of OCP efficacy [94]. However, in recent years, different types of combined hormone therapies have been identified and used to decrease natural steroid hormone release in the body. Drug innovation has allowed for a novel form of progestin to be combined with existing OCPs to maximize somatic symptom relief as well as the mood component. One such drug is a newer combined OCP containing nomegestrol acetate and 17-beta estradiol. A study completed by Robertson et al. (2021) found that 35 women (74.5%) reported positive mood changes to the drug, and only 10 (20.4%) women reported negative side effects. This preliminary study supports the effectiveness of this drug in the treatment of PMDD as an alternative to SSRIs [95]. Utilizing the same logic, another combined oral contraceptive contains drospirenone, a derivative of spironolactone, shown to have anti-mineralocorticoid and anti-androgenic activity. This combined with estradiol has been tested in multiple studies where PMDD symptoms were shown to improve. However, some of the studies noted a significant placebo effect, so larger randomized placebo-controlled trials are needed to confirm the efficacy of this drug [96].

OCPs are known to have numerous side effects, and can potentially lead to discontinuation of the medication among women. The known serious side effects include venous thrombosis, cardiovascular events, systemic lupus erythematosus, inflammatory bowel disease, breast cancer, and cervical cancer. OCP use is also associated with a higher risk for multiple sclerosis, bone fractures, increased body fat percentage and decreased lean mass, and urogenital problems. Depot medroxyprogesterone acetate injections have been associated with significantly higher human immunodeficiency virus (HIV) transmission as well as depressive symptoms [97, 98]. COCPs have been associated with a 25% increase in risk of depression for women below the age of 25, especially within 6 months of starting treatment. The relative risk of suicide attempts in adolescents and young women is also increased with hormonal contraceptive use, especially within 2 months of initiating treatment, with a doubling of relative risk for suicide attempts with OCPs and almost quadrupling with patches [97, 99]. Evidence suggests that women with underlying psychiatric illnesses such as depression or anxiety may be at higher risk of experiencing psychological adverse effects with OCP use [100]. One study found that 43.6% of participants overall, and 61.2% of participants with a history of psychiatric illness, reported experiencing mood changes with hormonal contraceptive use [101]. OCP use may also cause FSD due to the urogenital side effects such as dyspareunia, which could further affect psychological well-being and sexual function [97]. Levonorgestrel exposure has also been linked to anxiety and sleep problems [102]. A study that looked at psychological adverse effects of various formulations of OCPs hypothesized that the progestogen component may be responsible for the development of depressive symptoms [103]. Age may play a crucial part, since evidence suggests that adolescents may be more prone to developing mood symptoms as a side effect of OCP use [104, 105].

Understanding the Effects of SSRI on FSD and PMDD

Since the official characterization of PMDD in the DSM-5 as a depressive disorder, therapeutics have been within reach for patients to manage their condition. As previously explored, this disorder has serious implications for the psychological, social, and physical aspects of one’s life. PMDD is a mood disorder, and most patients have lower serotonin levels in the brain when experiencing symptoms [106]. Antidepressants and selective serotonin reuptake inhibitors (SSRIs) have been most commonly recommended as treatment options for patients experiencing PMDD [9]. SSRIs work by inhibiting the reuptake of serotonin in the brain, allowing the serotonin molecule to remain in the synapse of the postsynaptic neuron longer, which leads to improved mood [106, 107]. Women with PMDD are known to have atypical serotonergic transmitters due to a decreased number of receptors, decreased plasma serotonin levels in the luteal menstrual phase, and higher levels in the follicular menstrual phase, contributing to the symptoms experienced by patients [92, 108, 109]. SSRIs have minor effects on other neurotransmitters such as norepinephrine or dopamine. Sertraline, fluoxetine, escitalopram, citalopram, and paroxetine are all used to treat PMDD with fairly similar efficacy, but as with most psychiatric drugs, it is based on the individual’s brain chemistry and their tolerance for a particular drug [107].

Patients with PMDD have shown marked improvement in symptoms with many cases of rapid mood changes. SSRIs are orally administered and must undergo first-pass drug metabolism; however, the rapid mood changes suggest a difference in drug-neuron interaction compared to a patient taking SSRIs for classic depression [108, 109]. This is likely due to SSRIs' unique property of upregulating neuroactive steroids like allopregnanolone (ALLO). This metabolite, synthesized from 5-alpha dihydroprogesterone, gets converted within minutes [106]. In women, progesterone metabolites accumulate in the brain due to their lipophilic nature and ability to cross the blood-brain barrier. Normally, higher levels are generally seen in the basal hypothalamus, substantia nigra, and amygdala of the brain. Mood symptoms experienced by women with PMDD are likely related to progesterone and ALLO levels in the brain. High levels of ALLO, as seen in pregnancy, appear to have a mood-stabilizing effect. This principle can be directly applied to PMDD as patients with mood changes are recognized to have lower ALLO levels in the brain. Lower levels stimulate the amygdala and induce stress responses like cortisol release, manifesting as undesirable moods. Administration of SSRIs upregulates ALLO production, thereby decreasing amygdala response and improving the patient’s mood [108].

However, not all patients respond to SSRI treatments, and it is estimated that almost half of the patients on its prescription stop after 6 months of use. Contrary to what was previously discussed, SSRIs may have a paradoxical effect on some individuals. Elevated levels of allopregnanolone can have adverse effects. 5α-reductase has been administered to inhibit allopregnanolone synthesis which showed reduced mood symptoms. In a randomized control study, 16 patients with PMDD and 16 controls were given 5α-reductase, and reduced irritability and mood swings were reported [108, 109]. There is more evidence proving higher levels of allopregnanolone in the brain are able to have better mood-stabilizing effects compared to inhibiting its synthesis as larger samples of the population and a greater number of confirmatory studies have been reviewed. As difficult as mood disorders with sexual dysfunction are to characterize, it is important to consider this paradoxical effect noted in the study [106].

Patients now have the option of intermittent dosing instead of long-term use of SSRIs. Intermittent dosing, also known as luteal phase dosing, reduces risks associated with long-term antidepressant use and withdrawal. It is also beneficial as patients are able to comply with the therapy for a longer time as it is easier to take the medication at the onset of symptoms as opposed to daily use. However, if a patient is experiencing greater, long-term depressive moods or other somatic symptoms, an SSRI course of greater duration may certainly be recommended [93, 106]. Luteal phase (intermittent) dosing involves administering SSRIs 14 days prior to menses or when symptoms are noted in the patient. Riley et al. performed a systematic review comparing intermittent and continuous dosing for efficacy. 8 studies were fully reviewed. All studies were conducted for a minimum of 2 months with one comparative group given intermittent dosing and the other continuous; no placebo group was used. No significant difference in response rates between intermittent and continuous dosing was seen in patients [109]. Results show there is no statistical significance in the treatment of patients with intermittent dosing versus continuous. Other comparative studies assessed anger or irritability in a patient's daily record of symptoms and problems (DRSP score) prior and post medication which also concurred the results with the meta-analysis study [107].

Despite the benefits SSRIs can have on patients with PMDD, there are negative implications we must consider with this therapeutic option. Common side effects of SSRI use in PMDD include insomnia, anxiety, headache, and nausea. SSRIs also have known long-term concerns regarding weight gain and sexual dysfunction [9, 107]. These effects have not been properly evaluated in patients with PMDD at this time.

Challenges With Therapeutics Modalities

Current standards often conflate PMS and PMDD as synonymous conditions. It is important to note that these conditions are not the same, and patients experiencing either or both of these conditions have differing symptoms. The main distinction is that PMDD is recognized as a more severe, sometimes debilitating extension of PMS. If we cannot characterize this effectively, there may be concerns about drug dosing. Long-term use of medications with improper drug dosing will naturally lead to negative effects and poor patient outcomes, which is not desirable. This can have negative impacts on patients' lives from biological, psychological, and social perspectives [1-4].

OCP use and prescription also pose certain challenges that need to be overcome. In many cultures, there is still a stigma associated with the use of OCPs, as women who take them are assumed to be promiscuous, and misinformation that it can cause infertility is rife [110, 111]. Even where such concerns are not a factor, women are often reluctant to take OCPs due to concerns about adverse effects [112]. Existing literature on OCPs shows conflicting, inconsistent data and does not appear to sufficiently reflect these concerns about adverse effects. This is an area where further research is warranted, as identifying such issues can potentially lead to the development of formulations that can eliminate these concerns. Women who cannot take estrogen-containing formulations are also underserved in both the PMDD and FSD domains, since many of the existing therapies are based on them [110-112].

Regarding the long-term effects of SSRIs on patients with PMDD, there are still many areas to consider. Since this disorder has been characterized more recently, there is no statistical analysis of the long-term effects of SSRI usage in this patient population. Further research is needed to challenge the current understanding of therapeutics administered for PMDD. Unfortunately, this limitation can only be overcome with time as new studies and trials investigate this area. New therapeutic advances are continuous, and despite SSRIs being the gold standard treatment at this time, complacency cannot occur. Mood disorders related to FSD need to continue to be explored [93, 106-112].

Future directions

It is imperative to establish clear guidelines for diagnosing PMS and PMDD to enable clinicians to make definite diagnoses and treat each disease accordingly. There is a profound necessity to further investigate the association between PMDD and FSD, understanding the potential bidirectionality of the relationship and how the conditions might amplify each other. It is important to note that these conditions are interrelated, and most women who experience one will likely have symptoms related to the other condition as well. This emphasizes the importance of studying sexual function specifically in women with PMDD to gain a deeper understanding of the shared pathophysiology and the potential for targeted treatments that can reduce the burden of both conditions.

Although effective treatments are currently available, depending on the severity of functional distress and symptoms of the patient, there is a dire need to devise individualized management plans. Despite having FDA-approved treatments in place, the side effects are significant and important to monitor, and finding safer medications or therapies to improve patient outcomes is essential. It is imperative to develop validated prospective questionnaires or diaries to enable women to monitor premenstrual symptoms and sexual function simultaneously. Such a tool would allow for more accurate measurement of these symptoms throughout the menstrual cycle and during treatment, thus enhancing research efforts and advancing clinical practices. Importantly, the article underlines the importance of identifying women with PMDD using prospective monitoring of symptoms for multiple cycles to create a more homogeneous study group and enable more precise research outcomes. Understanding treatable contributing factors is crucial for devising targeted interventions that could improve the quality of life for women suffering from PMDD and FSD. It can then be said that despite current advancements in the understanding of PMDD and FSD, there is still much to be explored and understood [9, 11, 66-71].

## Conclusions

PMDD, a severe form of premenstrual syndrome, prominently features mood disturbances that have a cyclic nature, profoundly affecting women's quality of life and potentially leading to sexual disturbances. Female sexual dysfunction, characterized by persistent problems in sexual desire, response, or pain, is significantly influenced by hormonal fluctuations, psychological stressors, and interpersonal issues exacerbated by PMDD. Currently, approved treatment options for PMDD include SSRIs and COCPs. On the FSD spectrum, the only approved therapies within the scope of this review are flibanserin and bremelanotide.

One possible reason for the dearth of research may be the lack of standardization of FSD screening and evaluation tools used in the literature. Efforts to develop standardized tools that simultaneously evaluate PMDD and FSD symptoms may be the key to managing these conditions more effectively. Therefore, a call to action for healthcare systems and policymakers is warranted to prioritize and facilitate this integrated approach, thereby transforming the standard of care for women suffering from PMDD and FSD.

## References

[1] Sundström-Poromaa I (2018). The menstrual cycle influences emotion but has limited effect on cognitive function. Vitam Horm.

[2] Takeda T (2023). Premenstrual disorders: premenstrual syndrome and premenstrual dysphoric disorder. J Obstet Gynaecol Res.

[3] Westberg L, Eriksson E (2008). Sex steroid-related candidate genes in psychiatric disorders. J Psychiatry Neurosci.

[4] Rapkin AJ, Akopians AL (2012). Pathophysiology of premenstrual syndrome and premenstrual dysphoric disorder. Menopause Int.

[5] Lanza di Scalea T, Pearlstein T (2019). Premenstrual dysphoric disorder. Med Clin North Am.

[6] Pearlstein T, Steiner M (2008). Premenstrual dysphoric disorder: burden of illness and treatment update. J Psychiatry Neurosci.

[7] Ekenros L, Bäckström T, Hirschberg AL, Fridén C (2019). Changes in premenstrual symptoms in women starting or discontinuing use of oral contraceptives. Gynecol Endocrinol.

[8] Morishita C, Inoue T, Honyashiki M (2022). Roles of childhood maltreatment, personality traits, and life stress in the prediction of severe premenstrual symptoms. Biopsychosoc Med.

[9] Hantsoo L, Epperson CN (2015). Premenstrual dysphoric disorder: epidemiology and treatment. Curr Psychiatry Rep.

[10] Wittchen H -U, Becker E, Lieb R, Krause P (2002). Prevalence, incidence and stability of premenstrual dysphoric disorder in the community. Psychol Med.

[11] Kleinstäuber M, Schmelzer K, Ditzen B, Andersson G, Hiller W, Weise C (2016). Psychosocial profile of women with premenstrual syndrome and healthy controls: a comparative study. Int J Behav Med.

[12] Petersen N, London ED, Liang L, Ghahremani DG, Gerards R, Goldman L, Rapkin AJ (2016). Emotion regulation in women with premenstrual dysphoric disorder. Arch Womens Ment Health.

[13] Petersen N, Ghahremani DG, Rapkin AJ, Berman SM, Liang L, London ED (2018). Brain activation during emotion regulation in women with premenstrual dysphoric disorder. Psychol Med.

[14] Susser LC, Parish S, Dumas E, Nappi RE (2023). Premenstrual dysphoric disorder and sexual function: a narrative review. Sex Med Rev.

[15] Halbreich U, Borenstein J, Pearlstein T, Kahn LS (2003). The prevalence, impairment, impact, and burden of premenstrual dysphoric disorder (PMS/PMDD). Psychoneuroendocrinology.

[16] Biggs WS, Demuth RH (2011). Premenstrual syndrome and premenstrual dysphoric disorder. Am Fam Physician.

[17] Strelow B (2023). Premenstrual dysphoric disorder. JAAPA.

[18] Hofmeister S, Bodden S (2016). Premenstrual syndrome and premenstrual dysphoric disorder. Am Fam Physician.

[19] Appleton SM (2018). Premenstrual syndrome: evidence-based evaluation and treatment. Clin Obstet Gynecol.

[20] Richards MA, Oinonen KA (2022). Psychometric properties of a DSM-5-based screening tool for women's perceptions of premenstrual symptoms. Psychol Rep.

[21] Itriyeva K (2022). Premenstrual syndrome and premenstrual dysphoric disorder in adolescents. Curr Probl Pediatr Adolesc Health Care.

[22] Lanza di Scalea T, Pearlstein T (2017). Premenstrual dysphoric disorder. Psychiatr Clin North Am.

[23] Reid RL, Soares CN (2018). Premenstrual dysphoric disorder: contemporary diagnosis and management. J Obstet Gynaecol Can.

[24] Ryu A, Kim TH (2015). Premenstrual syndrome: a mini review. Maturitas.

[25] Younes Y, Hallit S, Obeid S (2021). Premenstrual dysphoric disorder and childhood maltreatment, adulthood stressful life events and depression among Lebanese university students: a structural equation modeling approach. BMC Psychiatry.

[26] Bunevicius R, Hinderliter AL, Light KC, Leserman J, Pedersen CA, Girdler SS (2005). Histories of sexual abuse are associated with differential effects of clonidine on autonomic function in women with premenstrual dysphoric disorder. Biol Psychol.

[27] Robinson RL, Swindle RW (2000). Premenstrual symptom severity: impact on social functioning and treatment-seeking behaviors. J Womens Health Gend Based Med.

[28] Braverman PK (2007). Premenstrual syndrome and premenstrual dysphoric disorder. J Pediatr Adolesc Gynecol.

[29] Zukov I, Ptácek R, Raboch J, Domluvilová D, Kuzelová H, Fischer S, Kozelek P (2010). Premenstrual dysphoric disorder--review of actual findings about mental disorders related to menstrual cycle and possibilities of their therapy. Prague Med Rep.

[30] Osborn E, Wittkowski A, Brooks J, Briggs PE, O'Brien PM (2020). Women's experiences of receiving a diagnosis of premenstrual dysphoric disorder: a qualitative investigation. BMC Womens Health.

[31] Delara M, Ghofranipour F, Azadfallah P, Tavafian SS, Kazemnejad A, Montazeri A (2012). Health related quality of life among adolescents with premenstrual disorders: a cross sectional study. Health Qual Life Outcomes.

[32] Gupta M, Dua D, Kaur H, Grover S (2019). Prevalence of premenstrual dysphoric disorder among school-going adolescent girls. Ind Psychiatry J.

[33] Mishra A, Banwari G, Yadav P (2015). Premenstrual dysphoric disorder in medical students residing in hostel and its association with lifestyle factors. Ind Psychiatry J.

[34] Cohen LS, Soares CN, Otto MW, Sweeney BH, Liberman RF, Harlow BL (2002). Prevalence and predictors of premenstrual dysphoric disorder (PMDD) in older premenopausal women. The Harvard Study of Moods and Cycles. J Affect Disord.

[35] Noviyanti NI, Gusriani Gusriani, Ruqaiyah Ruqaiyah, Mappaware NA, Ahmad M (2021). The effect of estrogen hormone on premenstrual syndrome (PMS) occurrences in teenage girls at Pesantren Darul Arqam Makassar. Gac Sanit.

[36] Rosenfeld R, Livne D, Nevo O, Dayan L, Milloul V, Lavi S, Jacob G (2008). Hormonal and volume dysregulation in women with premenstrual syndrome. Hypertension.

[37] Yen JY, Lin HC, Lin PC, Liu TL, Long CY, Ko CH (2019). Early- and late-luteal-phase estrogen and progesterone levels of women with premenstrual dysphoric disorder. Int J Environ Res Public Health.

[38] Lu D, Aleknaviciute J, Bjarnason R, Tamimi RM, Valdimarsdóttir UA, Bertone-Johnson ER (2021). Pubertal development and risk of premenstrual disorders in young adulthood. Hum Reprod.

[39] Freeman EW, Rickels K, Schweizer E, Ting T (1995). Relationships between age and symptom severity among women seeking medical treatment for premenstrual symptoms. Psychol Med.

[40] Rapkin AJ, Mikacich JA (2013). Premenstrual dysphoric disorder and severe premenstrual syndrome in adolescents. Paediatr Drugs.

[41] Basson R, Gilks T (2018). Women's sexual dysfunction associated with psychiatric disorders and their treatment. Womens Health (Lond).

[42] Faubion SS, Rullo JE (2015). Sexual dysfunction in women: a practical approach. Am Fam Physician.

[43] Weinberger JM, Houman J, Caron AT, Anger J (2019). Female sexual dysfunction: a systematic review of outcomes across various treatment modalities. Sex Med Rev.

[44] Wheeler LJ, Guntupalli SR (2020). Female sexual dysfunction: pharmacologic and therapeutic interventions. Obstet Gynecol.

[45] Clayton AH, Valladares Juarez EM (2019). Female sexual dysfunction. Med Clin North Am.

[46] Kingsberg SA, Clayton AH, Pfaus JG (2015). The female sexual response: current models, neurobiological underpinnings and agents currently approved or under investigation for the treatment of hypoactive sexual desire disorder. CNS Drugs.

[47] Parish SJ, Goldstein AT, Goldstein SW (2016). Toward a more evidence-based nosology and nomenclature for female sexual dysfunctions-part II. J Sex Med.

[48] Krakowsky Y, Grober ED (2018). A practical guide to female sexual dysfunction: an evidence-based review for physicians in Canada. Can Urol Assoc J.

[49] Nappi RE, Cucinella L, Martella S, Rossi M, Tiranini L, Martini E (2016). Female sexual dysfunction (FSD): Prevalence and impact on quality of life (QoL). Maturitas.

[50] Lin H, Fu HC, Wu CH, Tsai YJ, Chou YJ, Shih CM, Ou YC (2022). Evaluation of sexual dysfunction in gynecologic cancer survivors using DSM-5 diagnostic criteria. BMC Womens Health.

[51] Sungur MZ, Gündüz A (2014). A comparison of DSM-IV-TR and DSM-5 definitions for sexual dysfunctions: critiques and challenges. J Sex Med.

[52] Parish SJ, Hahn SR, Goldstein SW (2019). The International Society for the Study of Women's Sexual Health Process of Care for the Identification of Sexual Concerns and Problems in Women. Mayo Clin Proc.

[53] Parish SJ, Cottler-Casanova S, Clayton AH, McCabe MP, Coleman E, Reed GM (2021). The evolution of the female sexual disorder/dysfunction definitions, nomenclature, and classifications: a review of DSM, ICSM, ISSWSH, and ICD. Sex Med Rev.

[54] Shifren JL, Monz BU, Russo PA, Segreti A, Johannes CB (2008). Sexual problems and distress in United States women: prevalence and correlates. Obstet Gynecol.

[55] Gregory A (2021). Understanding female sexual dysfunction, its causes and treatments. Br J Nurs.

[56] Latif EZ, Diamond MP (2013). Arriving at the diagnosis of female sexual dysfunction. Fertil Steril.

[57] Frank JE, Mistretta P, Will J (2008). Diagnosis and treatment of female sexual dysfunction. Am Fam Physician.

[58] Phillips NA (2000). Female sexual dysfunction: evaluation and treatment. Am Fam Physician.

[59] Chen CH, Lin YC, Chiu LH, Chu YH, Ruan FF, Liu WM, Wang PH (2013). Female sexual dysfunction: definition, classification, and debates. Taiwan J Obstet Gynecol.

[60] Imprialos KP, Koutsampasopoulos K, Katsimardou A, Bouloukou S, Theodoulidis I, Themistoklis M, Doumas M (2021). Female sexual dysfunction: a problem hidden in the shadows. Curr Pharm Des.

[61] Jha S, Thakar R (2010). Female sexual dysfunction. Eur J Obstet Gynecol Reprod Biol.

[62] Levin RJ, Both S, Georgiadis J, Kukkonen T, Park K, Yang CC (2016). The physiology of female sexual function and the pathophysiology of female sexual dysfunction (committee 13a). J Sex Med.

[63] Cagnacci A, Venier M, Xholli A, Paglietti C, Caruso S (2020). Female sexuality and vaginal health across the menopausal age. Menopause.

[64] Bradway C, Boullata J (2014). Pharmacologic therapy for female sexual dysfunction. Nurse Pract.

[65] İlhan G, Verit Atmaca FV, Kurek Eken M, Akyol H (2017). Premenstrual syndrome is associated with a higher frequency of female sexual difficulty and sexual distress. J Sex Marital Ther.

[66] Conzatti M, Maciel RF, Perez AV, De Castro DH, Sbaraini M, Wender MC (2021). Premenstrual syndrome and female sexual function. J Sex Marital Ther.

[67] Wood C, Larsen L, Williams R (1979). Social and psychological factors in relation to premenstrual tension and menstrual pain. Aust N Z J Obstet Gynaecol.

[68] Winter EJ, Ashton DJ, Moore DL (1991). Dispelling myths: a study of PMS and relationship satisfaction. Nurse Pract.

[69] Nowosielski K, Drosdzol A, Skrzypulec V, Plinta R (2010). Sexual satisfaction in females with premenstrual symptoms. J Sex Med.

[70] Yang M, Gricar JA, Maruish ME, Hagan MA, Kornstein SG, Wallenstein GV (2010). Interpreting Premenstrual Symptoms Impact Survey scores using outcomes in health-related quality of life and sexual drive impact. J Reprod Med.

[71] Brotto L, Atallah S, Johnson-Agbakwu C (2016). Psychological and interpersonal dimensions of sexual function and dysfunction. J Sex Med.

[72] Marjoribanks J, Brown J, O'Brien PM, Wyatt K (2013). Selective serotonin reuptake inhibitors for premenstrual syndrome. Cochrane Database Syst Rev.

[73] Lodise NM (2017). Female sexual dysfunction: a focus on flibanserin. Int J Womens Health.

[74] Kingsberg SA, Clayton AH, Portman D (2019). Bremelanotide for the treatment of hypoactive sexual desire disorder: two randomized phase 3 trials. Obstet Gynecol.

[75] Edinoff AN, Sanders NM, Lewis KB, Apgar TL, Cornett EM, Kaye AM, Kaye AD (2022). Bremelanotide for treatment of female hypoactive sexual desire. Neurol Int.

[76] Kroll R, Archer DF, Lin Y, Sniukiene V, Liu JH (2018). A randomized, multicenter, double-blind study to evaluate the safety and efficacy of estradiol vaginal cream 0.003% in postmenopausal women with dyspareunia as the most bothersome symptom. Menopause.

[77] Da Silva AS, Baines G, Araklitis G, Robinson D, Cardozo L (2021). Modern management of genitourinary syndrome of menopause. Fac Rev.

[78] Shin JJ, Kim SK, Lee JR, Suh CS (2017). Ospemifene: a novel option for the treatment of vulvovaginal atrophy. J Menopausal Med.

[79] Pearlstein T (2016). Treatment of premenstrual dysphoric disorder: therapeutic challenges. Expert Rev Clin Pharmacol.

[80] Sundström-Poromaa I, Comasco E (2023). New pharmacological approaches to the management of premenstrual dysphoric disorder. CNS Drugs.

[81] Razali NA, Sidi H, Choy CL, Roos NA, Baharudin A, Das S (2022). The role of bupropion in the treatment of women with sexual desire disorder: a systematic review and meta-analysis. Curr Neuropharmacol.

[82] Rapkin AJ, Korotkaya Y, Taylor KC (2019). Contraception counseling for women with premenstrual dysphoric disorder (PMDD): current perspectives. Open Access J Contracept.

[83] Cappelletti M, Wallen K (2016). Increasing women's sexual desire: The comparative effectiveness of estrogens and androgens. Horm Behav.

[84] Stewart M, Black K (2015). Choosing a combined oral contraceptive pill. Aust Prescr.

[85] Golobof A, Kiley J (2016). The current status of oral contraceptives: progress and recent innovations. Semin Reprod Med.

[86] Both S, Lew-Starowicz M, Luria M (2019). Hormonal contraception and female sexuality: position statements from the European Society of Sexual Medicine (ESSM). J Sex Med.

[87] McCool-Myers M, Theurich M, Zuelke A, Knuettel H, Apfelbacher C (2018). Predictors of female sexual dysfunction: a systematic review and qualitative analysis through gender inequality paradigms. BMC Womens Health.

[88] Caruso S, Palermo G, Caruso G, Rapisarda AM (2022). How does contraceptive use affect women's sexuality? A novel look at sexual acceptability. J Clin Med.

[89] de Castro Coelho F, Barros C (2019). The Potential of Hormonal Contraception to Influence Female Sexuality. Int J Reprod Med.

[90] Čiaplinskienė L, Žilaitienė B, Verkauskienė R, Žalinkevičius R, Bumbulienė Ž, Vanagienė V, Bitzer J (2016). The effect of a drospirenone-containing combined oral contraceptive on female sexual function: a prospective randomised study. Eur J Contracept Reprod Health Care.

[91] Hantsoo L, Epperson CN (2020). Allopregnanolone in premenstrual dysphoric disorder (PMDD): Evidence for dysregulated sensitivity to GABA-A receptor modulating neuroactive steroids across the menstrual cycle. Neurobiol Stress.

[92] Tiranini L, Nappi RE (2022). Recent advances in understanding/management of premenstrual dysphoric disorder/premenstrual syndrome. Fac Rev.

[93] Stefaniak M, Dmoch-Gajzlerska E, Jankowska K, Rogowski A, Kajdy A, Maksym RB (2023). Progesterone and its metabolites play a beneficial role in affect regulation in the female brain. Pharmaceuticals (Basel).

[94] Lete I, Lapuente O (2016). Contraceptive options for women with premenstrual dysphoric disorder: current insights and a narrative review. Open Access J Contracept.

[95] Robertson E, Thew C, Thomas N, Karimi L, Kulkarni J (2021). Pilot data on the feasibility and clinical outcomes of a nomegestrol acetate oral contraceptive pill in women with premenstrual dysphoric disorder. Front Endocrinol (Lausanne).

[96] Breech LL, Braverman PK (2010). Safety, efficacy, actions, and patient acceptability of drospirenone/ethinyl estradiol contraceptive pills in the treatment of premenstrual dysphoric disorder. Int J Womens Health.

[97] Williams WV, Brind J, Haynes L (2021). Hormonally active contraceptives part I: risks acknowledged and unacknowledged. Linacre Q.

[98] Civic D, Scholes D, Ichikawa L (2000). Depressive symptoms in users and non-users of depot medroxyprogesterone acetate. Contraception.

[99] Hughes LD, Majekodunmi O (2018). Hormonal contraception and suicide: a new dimension of risk. Br J Gen Pract.

[100] Schaffir J, Worly BL, Gur TL (2016). Combined hormonal contraception and its effects on mood: a critical review. Eur J Contracept Reprod Health Care.

[101] Martell S, Marini C, Kondas CA, Deutch AB (2023). Psychological side effects of hormonal contraception: a disconnect between patients and providers. Contracept Reprod Med.

[102] Slattery J, Morales D, Pinheiro L, Kurz X (2018). Cohort study of psychiatric adverse events following exposure to levonorgestrel-containing intrauterine devices in UK general practice. Drug Saf.

[103] Mu E, Kulkarni J (2022). Hormonal contraception and mood disorders. Aust Prescr.

[104] Lundin C, Wikman A, Bixo M, Gemzell-Danielsson K, Sundström Poromaa I (2021). Towards individualised contraceptive counselling: clinical and reproductive factors associated with self-reported hormonal contraceptive-induced adverse mood symptoms. BMJ Sex Reprod Health.

[105] Johansson T, Vinther Larsen S, Bui M, Ek WE, Karlsson T, Johansson Å (2023). Population-based cohort study of oral contraceptive use and risk of depression. Epidemiol Psychiatr Sci.

[106] Pearlstein T (2002). Selective serotonin reuptake inhibitors for premenstrual dysphoric disorder: the emerging gold standard?. Drugs.

[107] Carlini SV, Deligiannidis KM (2020). Evidence-based treatment of premenstrual dysphoric disorder: a concise review. J Clin Psychiatry.

[108] Sundström-Poromaa I, Comasco E, Sumner R, Luders E (2020). Progesterone - Friend or foe?. Front Neuroendocrinol.

[109] Reilly TJ, Wallman P, Clark I, Knox CL, Craig MC, Taylor D (2023). Intermittent selective serotonin reuptake inhibitors for premenstrual syndromes: a systematic review and meta-analysis of randomised trials. J Psychopharmacol.

[110] Adongo PB, Tabong PT, Azongo TB, Phillips JF, Sheff MC, Stone AE, Tapsoba P (2014). A comparative qualitative study of misconceptions associated with contraceptive use in southern and northern ghana. Front Public Health.

[111] Meurice ME, Otieno B, Chang JJ, Makenzius M (2021). Stigma surrounding contraceptive use and abortion among secondary school teachers: A cross-sectional study in Western Kenya. Contracept X.

[112] Chen CX, Shieh C, Draucker CB, Carpenter JS (2018). Reasons women do not seek health care for dysmenorrhea. J Clin Nurs.

